# Predicting local adaptation in fragmented plant populations: implications for restoration genetics

**DOI:** 10.1111/j.1752-4571.2012.00284.x

**Published:** 2012-07-19

**Authors:** Melinda Pickup, David L Field, David M Rowell, Andrew G Young

**Affiliations:** 1 CSIRO Plant IndustryCanberra, ACT, Australia; 2 Division of Evolution, Ecology and Genetics, Research School of Biology, Australian National UniversityCanberra, ACT, Australia

**Keywords:** adaptive differentiation, environmental heterogeneity, *F*_ST_, population size, *Q*_ST_, *Rutidosis leptorrhynchoides*

## Abstract

Understanding patterns and correlates of local adaptation in heterogeneous landscapes can provide important information in the selection of appropriate seed sources for restoration. We assessed the extent of local adaptation of fitness components in 12 population pairs of the perennial herb *Rutidosis leptorrhynchoides* (Asteraceae) and examined whether spatial scale (0.7–600 km), environmental distance, quantitative (*Q*_ST_) and neutral (*F*_ST_) genetic differentiation, and size of the local and foreign populations could predict patterns of adaptive differentiation. Local adaptation varied among populations and fitness components. Including all population pairs, local adaptation was observed for seedling survival, but not for biomass, while foreign genotype advantage was observed for reproduction (number of inflorescences). Among population pairs, local adaptation increased with *Q*_ST_ and local population size for biomass. *Q*_ST_ was associated with environmental distance, suggesting ecological selection for phenotypic divergence. However, low *F*_ST_ and variation in population structure in small populations demonstrates the interaction of gene flow and drift in constraining local adaptation in *R. leptorrhynchoides*. Our study indicates that for species in heterogeneous landscapes, collecting seed from large populations from similar environments to candidate sites is likely to provide the most appropriate seed sources for restoration.

## Introduction

Habitat loss and fragmentation has led to the increasing need for genetic rescue of small or declining plant populations. A central and often controversial issue for population augmentation or restoration is the choice of appropriate source material (e.g. Broadhurst et al. 2008). Transplanting foreign genotypes with lower fitness than local genotypes can have important implications for the success of restoration efforts and the long-term viability of restored populations (Helenurm 1998; Galloway and Fenster 2000; Montalvo and Ellstrand 2000; Hufford and Mazer 2003). Currently, seed sourcing guidelines advocate the use of ‘local’ populations to minimize the risk of disrupting locally adapted genotypes. However, these guidelines are based on the assumption that plant species show adaptive differentiation and that this scales with geographic distance. To date, most studies of local adaptation have involved comparisons of populations over steep environmental gradients (latitude or altitude) or from contrasting habitat types (e.g. Bennington and McGraw 1995; Nagy and Rice 1997; Wright et al. 2006), which increases the likelihood of detecting local adaptation. In comparison, few studies have examined broad scale patterns of local adaptation in relation to spatial scale (as a surrogate of environmental variation and genetic divergence; Galloway and Fenster 2000; Joshi et al. 2001; Becker et al. 2006) or environmental heterogeneity (Montalvo and Ellstrand 2000; Raabová et al. 2007; Hereford and Winn 2008). Considering that seed sourcing decisions are often based on geographic proximity, and the broad distribution of many plant species in heterogeneous landscapes, the choice of appropriate genetic material for restoration requires an understanding of patterns of adaptation across a range of scales. Moreover, two recent meta-analyses (Leimu and Fischer 2008; Hereford 2009) found evidence of local adaptation, but that in many cases, it was either not present or the foreign genotypes outperformed local ones. This challenges the common assumption of local adaptation and indicates the need to examine adaptive differentiation across a broad range of environments and spatial scales to provide appropriate guidelines for restoration genetics.

Predicting and understanding the processes underlying local adaptation is a major challenge in restoration genetics. Variation in local adaptation among populations results from the interplay of selection, gene flow and stochastic processes (genetic drift and mutation). Depending on the strength of selection, gene flow may homogenize populations and constrain the development of local adaptation (Slatkin 1987; Kawecki and Ebert 2004; Sambatti and Rice 2006), while drift becomes more important as population size declines (Barrett and Kohn 1991). Gene flow may have a greater effect on local adaptation in small populations (Holt and Gomulkiewicz 1997; Jakobsson and Dinnetz 2005), and for species with high effective migration rates (e.g. self-incompatible species; Schierup et al. 2000; Castric and Vekemans 2004). High rates of gene flow combined with heterogeneous environments in some species may also select for phenotypic plasticity rather than locally adapted genotypes (Sultan and Spencer 2002). Understanding the interaction and relative importance of natural selection, drift and gene flow can therefore offer valuable insights into patterns of adaptation in heterogeneous landscapes and provide important data for informing plant translocation or restoration efforts.

Variation in the scale of local adaptation (reviewed in Linhart and Grant 1996) provides a challenge in delineating seed sourcing zones for restoration. At smaller scales where gene flow is higher, stronger local selection is required to overcome the homogenizing effects of gene flow (e.g. Antonovics and Bradshaw 1970; Sambatti and Rice 2006). Consequently, environmental distance, a measure of the ecological differences among populations, may be a better predictor of local adaptation than geographic distance (Montalvo and Ellstrand 2001). Comparing population differentiation for quantitative traits (*Q*_ST_) (Spitze 1993) and neutral genetic markers (*F*_ST_) (Wright 1951) can also provide information on the relative importance of selection, drift and gene flow for patterns of local adaptation (Leinonen et al. 2008; Whitlock 2008). However, to our knowledge, no studies have combined these predictive matrices (spatial scale, environmental distance, *F*_ST_ and *Q*_ST_) with transplant experiments to examine the relative importance of selection and gene flow for patterns of local adaptation. Accordingly, knowing the relative predictive power of geographic distance, environmental distance, *Q*_ST_ and *F*_ST_ can provide information on the key variables to consider when choosing seed sources for restoration, particularly in heterogeneous landscapes.

Understanding the role of population size in determining patterns of adaptive population differentiation is particularly relevant to plant restoration and management. Small population size has been associated with reduced genetic variation and plant fitness (Young et al. 1996; Leimu et al. 2006) and lower adaptive potential (Willi et al. 2006). When considering patterns of adaptive differentiation, Leimu and Fischer (2008) found that local adaptation was less prevalent in small compared with large populations. Reduced local adaptation in small populations may be associated with lower genetic variation (Stockwell et al. 2003; Willi et al. 2007), reduced efficacy of selection relative to genetic drift (Weber and Diggins 1990) and greater inbreeding (Keller and Waller 2002). Additionally, asymmetries in gene flow from large to small populations may result in the swamping of locally adapted genotypes in small populations (Holt and Gomulkiewicz 1997). This suggests that the size of the local and foreign population may influence patterns of local adaptation, and that population size can provide an important predictor when considering translocation among populations.

A reciprocal comparison of local and foreign plants in each habitat enables the most rigorous test of local adaptation (Turesson 1922; Clausen et al. 1940; Kawecki and Ebert 2004). However, reciprocal transplant experiments are usually only feasible for a small number of populations, which limits their usefulness in generalizing across populations, and for examining among-population variation in adaptation in relation to restoration genetics. This problem can be overcome by comparing pairs of local and foreign plants replicated across a broad range of spatial scales, environmental heterogeneity and population sizes. We used this approach to compare the performance of local (home-site) and foreign (non-local, immigrant) populations in 12 population pairs of the perennial herb *Rutidosis leptorrhynchoides* distributed across a large geographical area (0.7–600 km) of fragmented grassland habitats in South-Eastern Australia. This design enabled us to assess patterns of local adaptation and examine whether geographic distance, environmental distance, quantitative (*Q*_ST_) and molecular genetic differentiation (*F*_ST_) and population size could predict patterns of local adaptation. In addition, we examined patterns of local adaptation for high elasticity traits identified as those having the greatest demographic importance. The aims of our study were to first examine patterns of local adaptation, then assess correlates of adaptive differentiation to provide information on the variables most likely to predict local adaptation for restoration genetics.

## Methods

### Species and population pairs

**Figure 1:**
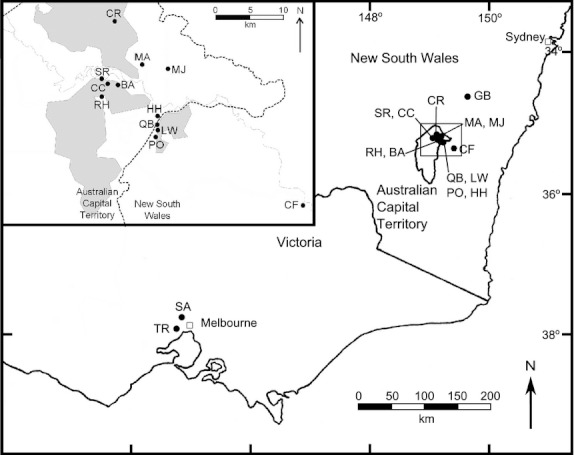
The geographic distribution of the 15 remnant populations of *Rutidosis leptorrhynchoides* across South-Eastern Australia. Each population is denoted by a closed circle and population code. Grey shading represents urban areas.

*Rutidosis leptorrhynchoides* F. Muell. (Asteraceae) is a herbaceous, insect-pollinated perennial with a sporophytic self-incompatibility system (Young et al. 2000a) that is endemic to highly fragmented temperate grasslands and grassy woodland communities in Australia. The species can live up to 20 years and has no long-term soil-stored seed bank (Morgan 1995a,b). The majority of *R. leptorrhynchoides* populations are smaller than 1000 plants, and the largest consists of ∼100 000 reproductive individuals. The 15 remaining diploid populations of *R. leptorrhynchoides* are distributed in two broad geographic zones; a northern zone in South-East New South Wales and the Australian Capital Territory (ACT), and a southern zone that extends through central Victoria (Fig. 1). To assess patterns of local adaptation, we chose 12 population pairs to span the geographic distribution of the species and to ensure that population pairs represented an even spread across a range of spatial scales from <1 to 600 km (the first population is the local and the second the foreign population (distance between populations, km); LW-QB (0.7), SR-CC (1.5), MA-BA (4.0), HH-MA (8.0), QB-RH (9.6), CR-LW (15.2), RH-CF (34.8), MJ-GB (71.9), GB-PO (78.9), SR-TR (506.2), CF-SA (516.0), GB-SA (575.1)). Because of the limited number of remaining *R. leptorrhynchoides* populations, site access restrictions and their uneven distribution in different geographic distance classes, some populations were used in multiple pairs. Population pairs were, however, selected to ensure replication at each distance class while minimizing the number of times a population was included (only GB and SR are used twice as a local population). While the use of populations in multiple pairs does raise issues associated with the assumption of independence, each pair is treated as independent and unique maternal families were used in each pair. We also correct the alpha level for the use of populations in multiple pairs. We assigned populations as either the local or foreign population in each pair to ensure that they were used as both origin types (local and foreign) and on the basis of site access restrictions (for soil collections, see below).

### Experimental design

This experiment was initially undertaken as a field trial (in 2003), but because of prevailing drought conditions, no plants survived past three months. Here, we present an alternative experimental approach that examined local adaptation in relation to soil and climate variables. For this design, we first defined the environmental components that differentiated sites to provide information on the variables that may drive patterns of local adaptation. We used multivariate analysis of bioclimatic and edaphic variables across all sites (see Environmental distance matrix) to identify the most likely environmental parameters distinguishing these populations. This approach identified two distinct climate zones that aligned with the North-South geographical divide of populations (Fig. S1a). However, we found that soil characteristics differentiated sites within each climate zone (Fig. S1b), and there was only a marginally significant association between soil distance and geographic distance (see results; *r* = 0.31, *P* = 0.084). This indicated that soil characteristics were more variable across a range of spatial scales and followed a mosaic pattern of environmental heterogeneity. Given these results, planting seed from local and foreign populations into soil from the local population, and growing them in a climate representative of the local site, provides an effective experimental framework in which to assess local adaptation to these variables. Accordingly, for each population pair, we collected soil from the local population, and plants from the local and foreign population were grown in local soil in a common climate representative of the northern climate zone at the Commonwealth Scientific Industrial Research Organisation (CSIRO) Plant Industry in Canberra, ACT (35°16′23″S, 149°06′42″E). The overall climate at this location is generally representative of the local population for all population pairs assessed because all local populations were from northern climate zone (Fig. S1a). Consequently, differences in soil were the main driver of environmental differences among the nine population pairs where the local and foreign populations were from the northern climate zone. For the three pairs where the foreign population was from the southern climate zone, both soil and climate differences contributed to environmental differences among sites.

### Predictors of local adaptation

#### Reproductive population size

We obtained reproductive population size for local and foreign populations by direct counts for populations with fewer than 10 000 plants. For larger populations, we estimated average reproductive plant density by counting the number of reproductive individuals in 3–6 quadrats (10 × 10 m or 30 × 30 m), and this was then multiplied by population area to determine total population size (see Pickup and Young 2008).

#### Environmental distance matrix

We used soil, climate and elevation to characterize the environment at each site and generate a composite measure of environmental distance between population pairs. We obtained climatic information on each site from the climate modelling program bioclim 3.14 (27 bioclimatic variables), elevation from GPS readings taken at each site and soil variables from soil composition and chemical soil analysis (16 variables). We constructed a correlation matrix for all 27 bioclimatic variables and separately for the 16 soil variables. Those with highly significant correlations (*P* < 0.001) were removed from the data set. The five least correlated bioclimatic variables (0.1 < *r* < 0.6) were elevation, highest period of radiation, mean temperature of wettest quarter, precipitation of the driest quarter and radiation of the driest quarter. The seven least correlated soil variables (0.01 < *r* < 0.7) were clay, coarse sand, copper, manganese, electrical conductivity, ammonium-nitrogen (NH_4_-N) and nitrate-nitrogen (NO_3_-N). A matrix of environmental distance was based on the Euclidean distance from the rotated factor scores for each population from the principle component analysis (PCA) for: (i) the five bioclimatic variables (two components, 80.3% of the variance explained), (ii) the seven soil variables (three components, 77.3% of the variance explained) and (iii) including all 12 bioclimatic and soil variables (three components, 70.1% of the variance explained).

#### Quantitative genetic distance matrix (*Q*_ST_)

We assessed quantitative genetic variation in growth and reproductive traits for 15 populations in a common garden (outdoor exclosure) using soil collected from native grassland and mixed in a ratio of 80:20 with river sand. Open-pollinated seed was collected from 1 to 3 inflorescences for 22–30 randomly chosen maternal plants in each population in December 2003–January 2004. For each maternal family from the 15 populations, 1–4 seed were planted into each of the three pots (0.5 L) in April 2004 (*n* = 3–12 seed per family). Pots were arranged in a complete randomized design. We scored germination and survival weekly for the first three months. At three months, all seedlings except the seedling closest to the geometric centre of the pot were removed. This experiment included 66–90 plants from each population (*n* = 1239). At 10 and 20 months, we measured the number of leaves (LVS), length of the longest leaf (LEN LF), width of the longest leaf (WD LF), plant height (HT), length of the longest stem (LEN ST), number of stems (ST), number of flowering stems (FL ST), proportion of flowering stems (PROP FL ST = FL ST/ST), number of inflorescences (INF) and floret number (FL; for 1–3 inflorescences). Given the high outcrossing rates in this species, progeny are more likely to be half- than full-sibs. However, correlated paternity scales with population size in this species, with the higher production of full-sib families in small populations (<100 plants) (see Young and Pickup 2010). We therefore estimated *Q*_ST_ using both half- and full-sib models, but given the very high correlation between estimates of *Q*_ST_ for half- and full-sib designs (*r* = 0.9968, *P* < 0.001), only results from the half-sib model are presented.

For each population pair (105 combinations) and trait [six least correlated traits (*r* = 0.02–0.69); LVS, LEN LF, LEN ST, ST, PROP FL ST, INF], we calculated *Q*_ST_ from variance components estimated using the ‘lme’ package and a script written for pairwise *Q*_ST_ comparisons (available on request) in r (Version 2.12.1). For the half-sib model (Spitze 1993): 
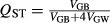
, where *V*_GB_ is the among-population variance and 4*V*_GW_ is the within-population genetic variance for half-sib families (Lynch and Walsh 1998). We estimated *V*_GW_ as the product of the trait heritability (*h*^2^) (average value from the heritability experiment, see Data S1 and Table S1) and within-population component of variance (*V*_W_): *V*_GW_ = *h*^2^*V*_W_. For each population pair, we calculated a pairwise *Q*_ST_ for each trait and then calculated an average *Q*_ST_ to provide an estimate of overall quantitative differentiation among populations and related this to patterns of local adaptation (Whitlock 2008).

#### Genetic distance matrix (*F*_ST_) and structure analysis

We used microsatellite markers to characterize genetic differentiation and admixture in populations of *R. leptorrhynchoides*. We collected leaf samples from all individuals from the quantitative genetics experiment (66–90 plants in each of 15 populations). Leaf samples were placed immediately in liquid N_2_, freeze dried at −80°C, and DNA was then extracted from 10 mg of ground leaf tissue according to the protocols of Blundell et al. (2010). One sample from each maternal family was randomly chosen for genotyping (22–28 individuals per population, *n* = 364). We used 10 primers from a microsatellite (SSR) library developed for *R. leptorrhynchoides* (RUT002, RUT004, RUT015, RUT34, RUT41; Savannah River Ecology Lab SSR development service and RUT359, RUT361, RUT372, RUT378, RUT384; M. Pickup unpublished, see Table S2).

For each of these primers, M13 inflorescence (Schuelke 2000) was used with M13 labelled SSR fragments amplified by PCR in a mixture of 5 μL containing: 2.7 μL H_2_0, 10x PCR buffer (Invitrogen, Waverley, Australia), 1.5 mm MgCl_2_, 0.25 μm universal fluorescent-labelled M13 primer, 0.05 μm forward primer (M13 tail), 0.25 μm reverse primer, 0.2 μm of each dNTP, 0.05 μL of 10% bovine serum albumin, 0.5 μL 10% PVP, 0.05 U of platinum Taq (Invitrogen) and 5 μL (25 ng) DNA template. Amplification of SSR fragments was performed on a Hybaid express thermocycler with a step-down PCR programme consisting of 94°C for 10 min; 15 cycles of 94°C for 30 s, 65°C for 30 s, 72°C for 1 min 20 s; 30 cycles of 94°C for 30 s, 50°C for 30 s, 72°C for 45 s. Amplified fragments were separated by capillary electrophoresis on an ABI 3130 Genetic Analyser (Applied Biosystems, Mulgrave, Australia), and genotypes were scored with GeneMapper v.4.0. We verified individual alleles manually, and samples with low or missing peaks were amplified and scored a second time.

We tested for deviations from Hardy–Weinberg and linkage equilibrium among loci using GDA (Lewis and Zaykin 2001), and null alleles and scoring errors using MICROCHECKER (van Oosterhout et al. 2004). We also used GDA to calculate the observed (*H*_O_) and expected heterozygosity (*H*_E_), number of effective alleles (*A*e) and inbreeding coefficient (*F*_IS_) for each of the 15 populations (see Table S3) and to estimate pairwise genetic differentiation and compare it to geographic distance [*F*_ST_/(1−*F*_ST_)] (Rousset 1997) (for 105 pairwise combinations).

We used the Bayesian clustering program structure (version 2.2) to assess population structure and assign individuals to *K* populations (Pritchard et al. 2000). We used the admixture model, no prior population information and correlated allele frequencies, for each of five replicates from *K* = 1 to *K* = 15. All analyses were run with 200 000 burn-in generations and 400 000 Markov Chain Monte Carlo. We used the replicates to assess the stability of independent estimates (average *r* > 0.98) and the most likely number of genetic clusters using the Δ*K* method (Evanno et al. 2005).

### Statistical analyses for distance matrices

We used Mantel tests to examine the correlation among the five distance matrices, (i) geographic distance, (ii) environmental distance (composite of soil and bioclimatic variables), (iii) soil distance, (iv) quantitative genetic distance (*Q*_ST_) and (v) molecular genetic distance (*F*_ST_). Mantel tests between each pair of distance matrices were performed using 10 000 permutations in the ‘ade4’ package in R (Version 2.12.1).

### Local adaptation experiment

#### Seed and soil collection

For all population pairs, we collected seed from one to three open-pollinated inflorescences from 27 to 80 maternal plants during January 2004. Fifteen maternal families were randomly chosen without replacement for each local and foreign population in each pair. This was to ensure that different maternal families were used for populations represented in multiple pairs. We collected soil (100–321 L) from multiple locations within each local population for each population pair and mixed these samples in a ratio of 80:20 with river sand (to facilitate drainage) and placed the mixture in 10 cm-diameter 0.5 L capacity pots.

#### Experimental planting

We randomly selected 12 seeds from each of the 15 open-pollinated families and weighed them in bulk on a four-decimal place gram balance. Seeds were then cold treated in a refrigerator set at ∼5°C for 72 h. For each family in each pair, we planted three seeds into each of four pots containing soil from the local population in May 2004. This gave *n* = 60 pots (15 families × 4 replicates) each for the local and foreign populations and a total *n* = 120 for each population pair. Across all population pairs *n* = 1440 pots and 4089 planted seed. We randomly paired each of the 15 maternal families from the local population with a maternal family from the foreign population. For each pair, this design resulted in 15 family comparisons of the relative performance of plants from the local and foreign populations (with four replicates per family). The four replicates of each family comparison (each replicate included one local and foreign plant) were then randomly allocated to the four blocks, so that each block contained one replicate. The position of the replicate in each block was arranged using a randomized row and column structure (10 rows × 27 columns). The four blocks were distributed across an outdoor enclosure. We supplemented natural precipitation with hand-watering every 1–2 days as required.

#### Fitness components

To compare the relative performance of local and foreign plants, we measured germination and seedling survival as well as two adult fitness components; number of inflorescences (reproduction; 12 and 24 months) and biomass (24 months). We recorded germination and survival weekly for the first three months. For pots where multiple seedlings had germinated, all seedlings except the one closest to the geometric centre of the pot were removed. Biomass samples were dried at 70°C for three days before weighing them on a four-decimal place gram balance. Previous demographic work for *R. leptorrhynchoides* (Young et al. 2000b) found that seedling and adult survivorship, as well as adult reproductive characteristics, had the highest elasticity values and therefore have a high contribution to population growth rate. Survival from seeding to adult was much higher in our experiment (>95%) compared to observations of plants in the field. Thus, in our experiment, we use biomass as a surrogate for adult survival, as plant size has been shown to be associated with survival in natural populations (A. G. Young, unpublished data).

#### The effect of origin (local or foreign) on plant growth and reproduction

To examine the effect of seed origin (local or foreign) on fitness components for all population pairs and comparing individual population pairs, we used (i) generalized linear mixed models for seedling survival (logistic regression: binomial distribution and logit link function) and number of inflorescences (Poisson distribution and log link function) and (ii) restricted maximum likelihood linear mixed models for seed weight and biomass. We examined the effect of origin, population pair and their interaction on each of the fitness-related traits. For these models, seed weight was fitted as a covariate and origin, population pair and the interaction between origin and population pair fitted as the main effects in the fixed model, while block, and row and column position within each block and maternal family (nested within population pair) were fitted in the random model. For seedling survival data were pooled for each maternal family. We then used the least significant difference (at α = 0.05) to assess whether there were significant differences between local and foreign plants in each of the 12 population pairs. For all analyses, nonsignificant terms (i.e. seed weight) were removed from the final model so that the simplest model is presented. To account for the analysis of three fitness components (seedling survival, biomass and number of inflorescences), we use an adjusted alpha value of 0.034 as outlined in García (2004). This adjustment takes into account the number of comparisons and correlations among the variables tested. The correlation among the three traits in our study was low (*r* = 0–0.17), but we chose the conservative adjusted value of 0.034 based on traits with *r* = 0.30 and the number of comparisons (*n*) = 5.

#### Linear regression analysis

We analysed the relation between the difference in fitness between local and foreign plants for each trait and (i) log local reproductive population size, (ii) log foreign reproductive population size, (iii) log geographic distance, (iv) environmental distance, (v) *Q*_ST_ and (vi) *F*_ST_, using multiple (and single) linear regressions. We used stepwise anova for model selection to identify the single variable, or combinations of variables that best explained the difference in fitness between local and foreign plants (lowest AIC value). Variables identified in the initial stepwise anova were subsequently analysed using simple or multiple linear regression. Given the number of population pairs (*n* = 12), a maximum of two explanatory variables were used in the multiple regression models. To account for the nonindependence of data points that shared a home population (i.e. SR-CC, SR-TR and GB-PO, GB-SA), we use a Bonferonni correction (alpha = 0.05/2) and test significance of these relations at alpha = 0.025. We also report these analyses using a subset of 10 population pairs (i.e. after the removal of the two most proximate population pairs, SR-CC and GB-PO) so that both SR and GB are used only once as a home population. We used Genstat 13th edition (VSN International, Oxford, UK) for all analyses.

## Results

### Predictor variables

The geographic distance matrix was significantly correlated with environmental distance based on soil and climate (*r* = 0.46, *P* = 0.029), but not with soil distance alone (*r* = 0.31, *P* = 0.084). The geographic distance matrix was also significantly correlated with *Q*_ST_ (*r* = 0.64, *P* = 0.0064), but not *F*_ST_ (*r* = −0.064, *P* > 0.05). We found a significant correlation between the quantitative genetic distance matrix (*Q*_ST_) and environmental distance based on both soil and climate (*r* = 0.61, *P* < 0.001) and soil alone (*r* = 0.51, *P* = 0.0092). In comparison, genetic distance based on molecular markers (*F*_ST_) and quantitative genetic distance (*Q*_ST_) were only marginally correlated (*r* = 0.44, *P* = 0.064) (see also Fig. S2).

### Population genetic structure

**Figure 2:**
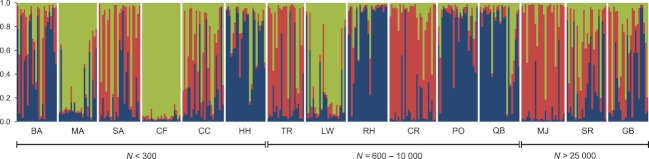
Results of the structure analysis for 15 populations of *Rutidosis leptorrhynchoides*. The optimal number of genetic clusters (*K*) is three following the ΔK method of Evanno et al. (2005). Each bar represents a single individual and its proportional membership to the three clusters. Populations are ordered by increasing population size from left to right.

Results from structure indicate a high degree of admixture in populations of *R. leptorrhynchoides* (Fig. 2). The Δ*K* method of Evanno et al. (2005) indicated that *K* = 3 is the most likely number of genetic clusters (Table S5). Most populations consisted of a mixture of the three clusters, while a number of small populations (*N* = 118–300) showed distinct genetic clustering with low levels of admixture (e.g. CF, MA). Moreover, there was greater variation in the level of admixture in small (<300) compared with large (>10 000) populations, with some small populations showing similar levels of admixture to the large populations (e.g. BA, CC; Fig. 2). The three genetic clusters were not associated with geographic proximity or environmental similarity (soils and climate).

### Patterns of local adaptation in fitness components

#### Seedling survival

We found a small but significant local genotype advantage for seedling survivorship (1.3 ± 0.4% survival) in the overall analysis (*P* = 0.004, Table 1), but the effect of origin varied among the population pairs (origin × population pair: *P* = 0.008, Table 1). Local adaptation was found in three of the 12 population pair comparisons (MA-BA, GB-PO and MJ-GB), with an increase in survival of 2.7–4.4% in the local population. None of the predictor variables explained variance among population pairs in patterns of local adaptation.

**Table 1:** Summary of the generalized linear mixed model and restricted maximum likelihood linear mixed model analyses to examine the effect of origin (local or foreign), population pair (Pop pair) and the interaction between origin and population pair (Origin × Pop pair) for seed weight and three fitness components in *Rutidosis leptorrhynchoides*

Fitness trait	Term	DF	*F*	*P*
Seed weight	Origin	1	1.93	0.167
	Pop pair	11	**6.20**	**<0.001**
	Origin × Pop pair	11	**3.64**	**<0.001**
Seedling survival	Origin	1	**8.37**	**0.004**
	Pop pair	11	1.56	0.109
	Origin × Pop pair	11	**2.35**	**0.008**
Biomass	Origin	1	0.31	0.578
	Pop pair	11	**111.97**	**<0.001**
	Origin × Pop pair	11	**2.68**	**0.002**
Number of inflorescences	Origin	1	**7.70**	**0.006**
	Pop pair	11	**20.68**	**<0.001**
	Origin × Pop pair	11	**2.59**	**0.003**

Significant terms (*P* < 0.034, corrected alpha value; see Methods) are highlighted in bold.

#### Adult biomass

**Figure 3:**
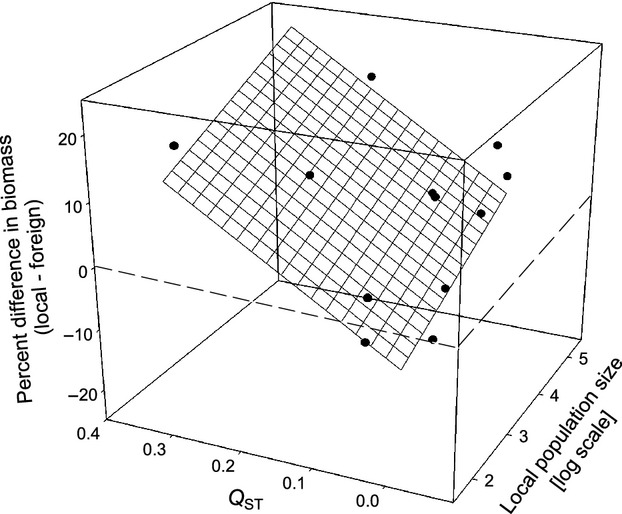
The percentage difference in mean biomass between the local and foreign populations (local – foreign) for 12 population pairs of *Rutidosis leptorrhynchoides* as a function of local reproductive population size (*x*) and *Q*_ST_ (*y*). Values above the dashed horizontal line (zero) represent local adaptation and those below represent foreign genotype advantage. The equation for the relation is: *z* = −20.03 + 4.17*x* + 68.2*y* (*R*^2^ = 0.46, *P* = 0.024).

We found no evidence of local adaptation for biomass in the overall analysis (origin: *P* > 0.05, Table 1), but there was a significant interaction between origin and population pair (origin × population pair: *P* = 0.002, Table 1). In three population pairs (SR-CC, RH-CF and GB-SA), there was evidence of local adaptation, with a percentage increase in biomass in the local population of 7.5–17.3%. Conversely, significant foreign genotype advantage was observed in LW-QB with 12.4% greater biomass in the foreign population in this pair. The degree of quantitative genetic differentiation (*Q*_ST_) and size of the home population explained 46.4% of the variance in local adaptation among population pairs (*P* = 0.024), with greater local adaption in population pairs with higher *Q*_ST_ and larger local population size (Fig. 3). This relation remained significant (*P* = 0.024) for the subset of 10 population pairs, with *Q*_ST_ and local population size explaining 55.5% of the variance in local adaptation.

#### Reproduction

We found a significant difference between local and foreign populations in the mean number of inflorescences (origin: *P* = 0.006, Table 1), but this varied among population pairs (origin × population pair: *P* = 0.003). Including all population pairs, there was evidence of significant foreign genotype advantage, with an average of 16.1% more inflorescences in the foreign population. Comparing individual population pairs, significant foreign genotype advantage was observed in GB-PO and SR-TR with an increase in the mean number of inflorescences in the foreign population of 47.1% and 75.8%, respectively. None of the predictor variables explained among population pair variance in the performance of local and foreign plants.

## Discussion

Our study demonstrates variation in patterns of adaptive differentiation among fragmented populations of the perennial plant *R. leptorrhynchoides*. There was little evidence of local adaptation across a range of fitness components with the equivalent performance of local and foreign genotypes in many population pairs. Where significant differences were encountered, the greater performance of foreign genotypes (foreign genotype advantage) was observed as frequently as local adaptation for many fitness components. For biomass, local and foreign genotype advantage was predicted by *Q*_ST_ and the size of the local population. Local adaptation was more evident in population pairs where *Q*_ST_ was high and where the local population was large, while foreign genotype advantage was more apparent in population pairs with low *Q*_ST_ and small local population size. We first discuss our results in the context of gene flow and patterns of environmental heterogeneity and consider the importance of population size for local adaptation. We then conclude with focussing on the importance of understanding patterns and predictors of adaptive differentiation for seed sourcing in restoration genetics.

### The importance of gene flow and environment for patterns of local adaptation

Understanding patterns of adaptation in relation to spatial scale, gene flow and environmental variation is important for determining appropriate seed sourcing zones for restoration. An association between adaptive population differentiation and spatial scale is expected if both genetic isolation and environmental heterogeneity increase with geographic distance (Galloway and Fenster 2000). In our study, there was no relation between geographic distance and local adaptation for any of the fitness components, with both local and foreign genotype advantage observed over a range of spatial scales from 0.7 to ∼600 km. Similarly, in other studies where local adaptation was observed, it was found not to scale with geographic distance (Montalvo and Ellstrand 2000; Raabová et al. 2007) or was only apparent at the largest distance classes (Galloway and Fenster 2000; Becker et al. 2006). Consequently, spatial scale may have limited value as a predictor of local adaptation and in the delineation of seed sourcing zones. The lack of association between local adaptation and geographic distance in our study is not surprising given the generally high levels of inferred gene flow among populations (low *F*_ST_) and admixture (Fig. 2). Mating system can influence gene flow, with less local adaptation expected in outcrossing species with higher rates of gene flow (Linhart and Grant 1996; but see Hereford 2010). Our data fit with this prediction given the self-incompatibility system of *R. leptorrhynchoides* which should increase effective migration rates (Castric and Vekemans 2004). The selective advantage of novel *S*-alleles may also increase the spread of immigrant alleles in populations, contributing to admixture and constraining local adaptation. Indeed, earlier studies (Pickup and Young 2008) have demonstrated increased fertilization success of inter-population cross pollinations compared with within-population crosses for this species.

In combination with gene flow, spatial patterns of environmental variation will determine adaptive differentiation, providing a challenge for the development of general seed sourcing guidelines. We found a mosaic pattern of environmental heterogeneity among populations of *R. leptorrhynchoides* (Figs S1 and S2). High gene flow and spatial environmental heterogeneity can select for plasticity over adaptation (Sultan and Spencer 2002; Thibert-Plante and Hendry 2011), so that low levels of adaptive differentiation and the occurrence of foreign genotype advantage may reflect the occurrence of phenotypic plasticity in *R. leptorrhynchoides*. Generalist genotypes and plasticity should also be favoured when there is temporal variability in selection pressures (Bradshaw 1965; Kawecki and Ebert 2004), such as in disturbance prone environments or in meta-populations with constant population turnover (Galloway and Fenster 2000). For populations of *R. leptorrhynchoides*, periodic disturbance by fire may result in temporal variability in selection regimes that would lead to less local adaptation and selection for generalist genotypes with broad ecological tolerance. Consequently, for species with limited local adaptation, a more important consideration for restoration genetics is likely to be sourcing from large, genetically diverse populations to maximize evolutionary potential.

A relation between the degree of local adaptation and environmental distance indicates the importance of environmental heterogeneity in driving patterns of adaptive differentiation. Environmental distance has been found to predict adaptive differentiation in previous plant studies (Montalvo and Ellstrand 2000; Raabová et al. 2007; Hereford and Winn 2008; Hereford 2009). The absence of an association between environmental distance and local adaptation in our study is surprising given the level of environmental differentiation between populations in soil characteristics and between the two climate zones (Fig. S1). We did, however, find an association between *Q*_ST_ and environmental distance that likely reflects that quantitative genetic differentiation has occurred in response to divergent selective pressures. For some populations, this might indicate that selection is strong enough to overcome the homogenizing effects of gene flow. Accordingly, in combination with population size, *Q*_ST_ predicted local adaptation for biomass, with greater local adaptation in population pairs with higher *Q*_ST_. The importance of *Q*_ST_ in explaining variation in patterns of local adaptation indicates that, in comparison with geographic distance or *F*_ST_, this matrix can be an important predictor of adaptive differentiation in heterogeneous landscapes.

### Does population size influence local adaptation?

Population size can influence patterns of adaptive differentiation by mediating the relative importance of selection and drift. We found greater local genotype advantage for biomass when the local population was large (and *Q*_ST_ was high). This may reflect the greater efficacy of selection in large populations (Linhart and Grant 1996) and was a key finding of Leimu and Fischer (2008) in their meta-analysis of local adaptation in plants. In small populations, several factors may reduce adaptive differentiation including the stochastic loss of beneficial alleles (Willi et al. 2007) or swamping out of locally adapted genotypes (Holt and Gomulkiewicz 1997). Moreover, with source-sink dynamics and asymmetrical dispersal rates (Kawecki and Holt 2002), selection will be biased towards the large source population so that less local adaptation is expected in small sink populations (e.g. Anderson and Geber 2010). Our results highlight the importance of demography and the interaction of drift and gene flow in structuring genetic variation. For example, small, isolated populations (e.g. CF, *N* = 210) showed genetic structure and had higher pairwise *F*_ST_ (0.07–0.1), while small populations closer to large ones (e.g. CC, *N* = 220) were highly admixed and had generally lower pairwise *F*_ST_ (0.03–0.07). Consequently, population size is an important consideration when choosing potential source populations for translocation and indicates that stochastic processes may play a central role in determining patterns of adaptation in fragmented populations.

## Some limitations of the study

One of the difficulties of measuring local adaptation in long-lived perennial species such as *R. leptorrhynchoides* is obtaining estimates of lifetime fitness. We found consistent patterns in the performance of local and foreign genotypes for the fitness components measured over 2 years (see Table S4), but long-term variation in fitness may influence fitness outcomes. Despite these limitations, we focussed on fitness components identified as high elasticity traits (see Young et al. 2000b) to ensure that our estimates of local adaptation were based on traits that are likely to have important demographic consequences. Using field-collected open-pollinated seed means that our results may reflect a combination of genotype and the seed maternal environment (Roach and Wulff 1987). Maternal effects are more likely to be important for early life history traits (Mousseau and Fox 1997), but in our study, the difference in performance of local and foreign populations did not decrease across the life cycle. Moreover, we used seed weight as a covariate to account for potential maternal effects and found that seed weight had little effect on seedling survival or adult growth and reproduction.

A number of environmental components may contribute to adaptive differentiation including soils (e.g. Snaydon and Davies 1982; Sambatti and Rice 2006), climate (e.g. Santamaria et al. 2003; Macel et al. 2007), vegetation composition (Raabová et al. 2007; Lawrence and Kaye 2011), competition (Kindell et al. 1996; Bischoff et al. 2006) and biotic interactions (Crémieux et al. 2008). For *R. leptorrhynchoides*, the presence of two climatic regions and a mosaic pattern of edaphic variation enabled us to examine local adaptation in relation to these variables by growing each population pair in the local soil and a climate representative of the local site. Furthermore, *Q*_ST_ was correlated with environmental distance (climate and soils), suggesting that these environmental variables are related to the selective pressures in each population. Although we were unable to assess the importance of other biotic or abiotic factors, our experimental framework enabled an assessment of local adaptation in relation to soil and climatic variation (northern versus southern climate zones), which, for this species, are likely important components of environmental heterogeneity across the landscape.

## Conclusions and implications for seed sourcing

Our study of local adaptation in *R. leptorrhynchoides* in relation to a number of predictive matrices and population characteristics has a number of implications for restoration genetics. Firstly, the importance of *Q*_ST_ and population size in predicting local adaptation indicates the role of selection and stochastic processes in determining patterns of adaptive differentiation (see also Hereford and Winn 2008). These results suggest that selecting seed from large, genetically diverse populations from similar environments is likely to provide the most appropriate seed sources for restoration. Secondly, geographic distance did not explain patterns of adaptation, suggesting that is not always a suitable surrogate for population differentiation in the delineation of seed sourcing zones. Finally, the absence of local adaptation and the superior performance of foreign genotypes in many population pairs indicate that for species with high levels of gene flow and spatial (and/or temporal) environmental heterogeneity, local adaptation may be limited. In this case, seed sourcing should focus on maximizing genetic diversity by sampling from large populations. Retaining genetic diversity in restored or augmented populations is especially important for species where diversity has a direct link to fitness (e.g. self-incompatible species; Young and Pickup 2010) and to maintain evolutionary potential in the face of global environmental change (Willi et al. 2006).

We observed substantial variation in patterns of adaptation among population pairs. This may reflect differences in population history, selection and drift and highlights the difficulties in generalizing from a small number of populations. Our results also suggest that predicting local adaptation and delineating seed sourcing zones may be more difficult for species that are distributed across mosaic environments compared to clinal variation or environmental gradients (e.g. altitude or latitude). Therefore, when considering seed sources based on adaptive differentiation, our results indicate that ecological differences among sites and the characteristics of the home and source population should form the basis of seed sourcing guidelines.

## Data archiving statement

Data for this study are available at Dryad: doi:10.5061/dryad.1qp8v.

## Supporting Information

**Figure S1.** The relationship between geographic distance and (a) climate distance and (b) soil distance for 12 pairs of populations of *Rutidosis leptorrhynchoides*.

**Figure S2.** The relationship between various distance matrices; (a) geographic distance and environmental distance, (b) geographic distance and soil distance, (c) geographic distance and *Q*_ST_, (d) geographic distance and *F*_ST_, (e) environmental distance and *Q*_ST_, (f) soil distance and *Q*_ST_ and (g) *Q*_ST_ and *F*_ST_, for 105 population pair comparisons (derived from 15 populations) of *Rutidosis leptorrhynchoides*.

**Table S1.** Heritability estimates from four populations for the seven traits used in the calculation of quantitative genetic differentiation among populations (*Q*_ST_).

**Table S2.** Microsatellite primers used to assess diversity and admixture in populations of *Rutidosis leptorrhynchoides* including the repeat unit sequence and allelic size range.

**Table S3.** Summary of the reproductive population size, genetic diversity measures and mean fixation (*F*_IS_) estimates in 15 populations of *Rutidosis leptorrhynchoides*.

**Table S4.** Repeated measures analysis to examine if the effect of plant origin (local or foreign) on progeny fitness varied between 12 and 24 months for the number of inflorescences including all population pairs.

**Table S5.** structure results to determine the most likely number of genetic clusters (*K*) following Evanno et al. (2005) for *Rutidosis leptorrhynchoides*.

**Data S1.** Materials and methods for the heritability experiment.
