# Supplementary material for: Predicting local adaptation in fragmented plant populations: implications for restoration genetics
Source: Evol Appl. 2012 Jul 19;5(8):913–24. doi: 10.1111/j.1752-4571.2012.00284.x (PMC3552408; doi:10.1111/j.1752-4571.2012.00284.x)
Supplement: Supplementary file 1 [file eva0005-0913-SD1.doc]

**Supporting information**


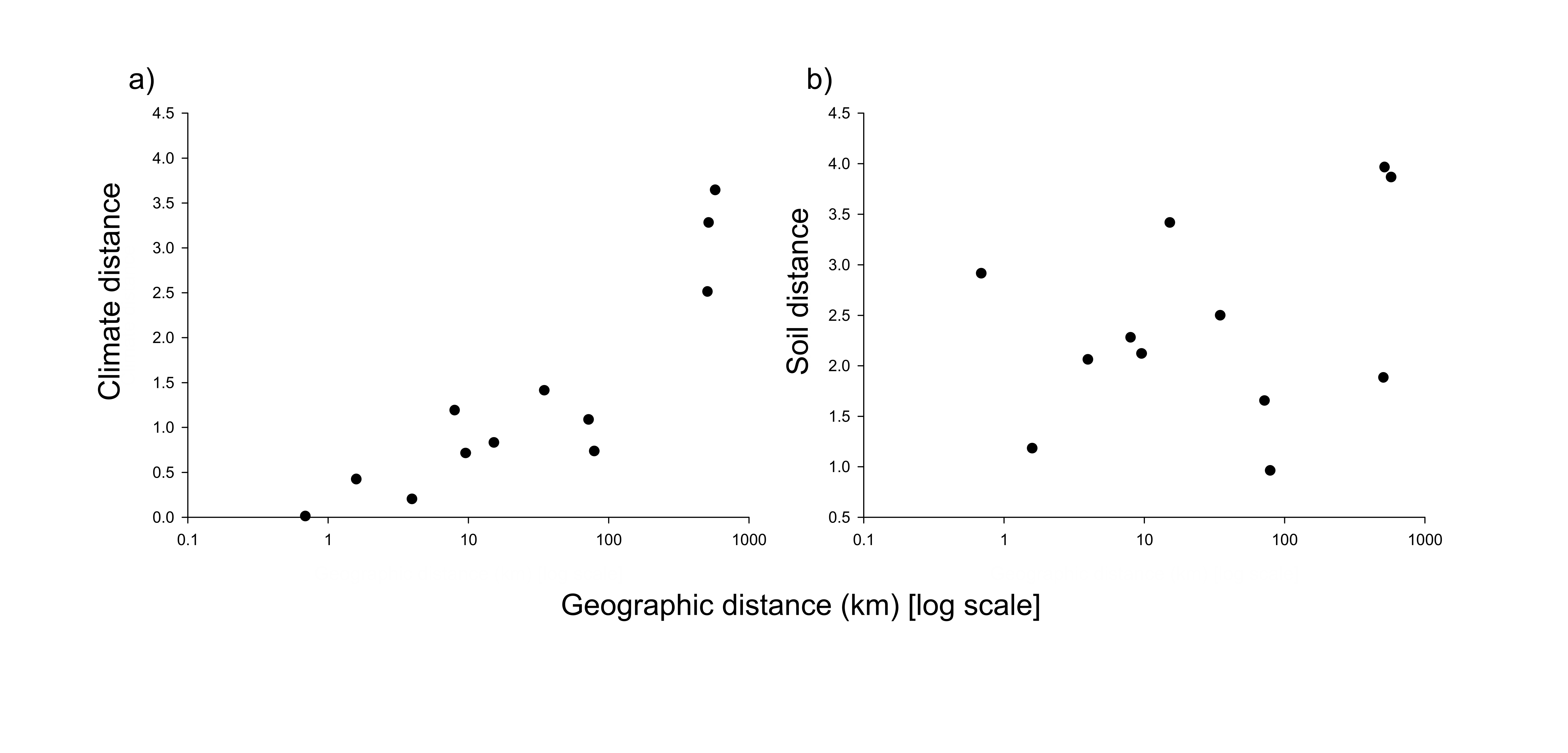


Figure S1. The relationship between geographic distance and (a) climate distance and (b) soil distance for 12 pairs of populations of *Rutidosis leptorrhynchoides*. Geographic distance is the linear distance (km) between populations in each pair. Climate and soil distance are the Euclidean distance between populations based on varimax rotated factor scores from Principle Components Analysis (PCA).

Figure S2. The relationship between various distance matrices; a) geographic distance and environmental distance, b) geographic distance and soil distance, c) geographic distance and *Q*_ST_, d) geographic distance and *F*_ST_, e) environmental distance and *Q*_ST_, f) soil distance and *Q*_ST_ and g) *Q*_ST_ and *F*_ST_, for 105 population pair comparisons (derived from 15 populations) of *Rutidosis leptorrhynchoides*. Open circles are the 12 population pairs used in this study, while filled circles are the remaining 93 population pairs. Geographic distance is the linear distance (km) (log scale) between populations in each pair. Environmental distance is the Euclidean distance between populations for both climate and soil variables, while soil distance is the Euclidean distance between populations based on varimax rotated factor scores from Principle Components Analysis (PCA).

Notes S1. Materials and methods for the heritability experiment

We established a common garden experiment to estimate trait heritability so that it could be included in our estimates of *Q*_ST_ for a range of growth and reproductive traits. Heritability was assessed in four populations of *R. leptorrhynchoides*; Letchworth, Goulburn, Majura and Stirling Ridge (reproductive population size > 1000) using a common garden with native grassland soil. Plants were located in the same outdoor exclosure as the local adaptation experiment. In December 2002 – January 2003, we sampled open pollinated seed from 1-3 inflorescences from 15 randomly chosen plants in each population. In March 2003, we planted three seed from each of the 15 maternal families into each of 12 pots (10cm 0.5L capacity) containing soil collected from local native grassland and mixed in a ratio of 80:20 with river sand (36 seed and 12 pots per family, *n* = 720). We weighed seed prior to planting to account for maternal effects. We were, however, unable to account for other potential maternal effects given that controlled crosses were not undertaken to produce half-sib families. We used a complete randomised block design with four blocks and three pots from each mother allocated to each block. We scored germination and survival weekly for the first three months. At three months all seedlings except the seedling closest to the geometric centre of the pot were removed. At 20 months we measured the number of leaves (LVS), length of the longest leaf (LEN LF), width of the longest leaf (WD LF), plant height (HT), length of the longest stem (LEN ST), number of stems (ST), number of flowering stems (FL ST), proportion of flowering stems (PROP FL ST = FL ST/ ST) and number of inflorescences (INF).

Trait heritability in each population can be estimated using variance components. Accordingly, for each population and trait, we performed an unbalanced ANOVA (Genstat 13th Edition, VSN International, Oxford UK) using the expected mean squares method to calculate variance components (PROP FL ST was arcsine transformed prior to analysis). Assuming that offspring within families were related as half sibs [(tm = 0.84 – 1; Young and Brown 1999) and low correlated paternity (rp < 0.5 for populations greater than 1000 plants; Young and Pickup 2010)], the narrow-sense heritability for each trait in each population was estimated as: $h^{2}=\frac{4\sigma_{f}^{2}}{\sigma_{f}^{2}+\sigma_{e}^{2}}$ , where $\sigma_{f}^{2}$ represents the family variance component and $\sigma_{e}^{2}$ the residual variance. An average heritability was then calculated for each trait (see supporting information, Table S1).

Table S1. Heritability estimates from four populations for the six traits used in the calculation of quantitative genetic differentiation among populations (*Q*_ST_). GB = Goulburn, LW = Letchworth, MJ = Majura, SR = Stirling Ridge.

|  | **Population** | | | |  |
| --- | --- | --- | --- | --- | --- |
| **Trait** | **GB** | **LW** | **MJ** | **SR** | **Mean** |
| Number of leaves (LVS) | 0.15 | 0.45 | 0.31 | 0.17 | 0.27 |
| Length of the longest leaf (LEN LF) | 0.45 | 0.1 | 0.78 | 0.18 | 0.38 |
| Length of the longest stem (LEN ST) | 0.33 | 0.05 | 0.37 | 0.47 | 0.31 |
| Number of stems (ST) | 0.06 | 0.08 | 0.09 | 0.19 | 0.11 |
| Proportion of flowering stems (PROP FL ST) | 0.24 | 0.19 | 0.24 | 0.78 | 0.36 |
| Number of inflorescences (INF) | 0.03 | 0.44 | 0.34 | 0.73 | 0.39 |

Table S2: Microsatellite primers used to assess diversity and admixture in populations of *Rutidosis leptorrhynchoides* including the repeat unit sequence and allelic size range.

| Locus | Locus name | Repeat motif | Primer sequence (5' - 3') | Size (bp) |
| --- | --- | --- | --- | --- |
| 1 | RUT002 | (AAAG)^6^ | TGTTGGCTTATCTAACTCTC | 299-341 |
|  |  |  | ATACTGCCAGAAGTAAAGTC |  |
| 2 | RUT004 | (ATC)^8^ | TTAAAAAGCATCCGTATATAG | 197-242 |
|  |  |  | CAATCGGCAAAGAGAC |  |
| 3 | RUT015 | (AGAT)^9^ | AAAAGATTAAGGGCTGATTA | 372-455 |
|  |  |  | CCATCCACGATAAAATAAA |  |
| 4 | RUT034 | (ACTC)^6^ | GGACGAGACGGATGGAAG | 191-295 |
|  |  |  | TTGGCCATCGGAGATGAAG |  |
| 5 | RUT041 | (AC)^11^ | GGATTCCACTCTTGATTATGTTATGC | 130-189 |
|  |  |  | CTTGATGTGTTCTTGAGTAGGTG |  |
| 6 | RUT359 | (GT)^14^ | GATAGCGGGAATGAGTTTAG | 190-239 |
|  |  |  | CGGGTCGGGAAAATAAGCAC |  |
| 7 | RUT361 | (GAA)^12^ | ATAACAGCACCGCCACC | 201-338 |
|  |  |  | TTAGCAAAATGGCCCATTCT |  |
| 8 | RUT372 | (CT)^18^ | CGATAATCCTTTTGGGTCACA | 203-296 |
|  |  |  | ACGTTATGAAGATGGGATATG |  |
| 9 | RUT378 | (GA)^21^ | AAGCAAATAGCGGGAATG | 178-228 |
|  |  |  | CCACCACAATCAATCGCCTTC |  |
| 10 | RUT384 | (GA)^12^ | GGTGGGTGTCATTACTCCATT | 125-189 |
|  |  |  | CCACCATACTCAAAACGTAGC |  |

Table S3: Summary of the reproductive population size, genetic diversity measures and mean fixation (*F*_IS_) estimates in 15 populations of *Rutidosis leptorrhynchoides*. *F*_IS_ was calculated using GDA (Lewis and Zaykin 2001). Abbreviations: Pop, Population; Pop size, reproductive population size; *N*, sample size; *A*, mean number of alleles per locus; *A*e, effective number of alleles; *H*_O_, observed heterozygosity; *H*_E_, expected heterozygosity; CI, confidence interval.

| **Pop** | **Pop size** | ***N*** | ***A*** | ***A*e** | ***H*_O_** | ***H*_E_** | ***F*_IS_** | **95% CI** |
| --- | --- | --- | --- | --- | --- | --- | --- | --- |
| GB | 95200 | 26 | 23.6 | 9.1 | 0.719 | 0.877 | 0.201 | 0.07-0.33 |
| CR | 4000 | 28 | 25.8 | 7.3 | 0.708 | 0.846 | 0.179 | 0.06-0.29 |
| SR | 69600 | 25 | 23.1 | 8.4 | 0.738 | 0.858 | 0.162 | 0.04-0.29 |
| LW | 1171 | 24 | 22.9 | 6.1 | 0.683 | 0.815 | 0.183 | 0.04-0.34 |
| RH | 3489 | 25 | 22.3 | 5.7 | 0.604 | 0.811 | 0.277 | 0.10-0.42 |
| CF | 210 | 25 | 21.8 | 4.5 | 0.700 | 0.760 | 0.103 | -0.05-0.27 |
| QB | 10000 | 24 | 22.9 | 6.8 | 0.710 | 0.825 | 0.161 | 0.02-0.30 |
| CC | 220 | 26 | 23.5 | 6.8 | 0.672 | 0.827 | 0.208 | 0.09-0.31 |
| MJ | 27626 | 26 | 24.2 | 7.0 | 0.674 | 0.841 | 0.219 | 0.05-0.37 |
| MA | 118 | 24 | 22.0 | 6.1 | 0.690 | 0.818 | 0.178 | 0.10-0.25 |
| HH | 300 | 25 | 22.2 | 7.2 | 0.713 | 0.827 | 0.161 | 0.00-0.31 |
| BA | 81 | 24 | 22.5 | 7.2 | 0.709 | 0.842 | 0.180 | 0.05-0.29 |
| PO | 8171 | 25 | 22.1 | 5.8 | 0.668 | 0.824 | 0.209 | 0.05-0.37 |
| SA | 137 | 26 | 23.8 | 8.1 | 0.762 | 0.839 | 0.113 | -0.01-0.23 |
| TR | 626 | 25 | 21.3 | 8.6 | 0.715 | 0.857 | 0.189 | 0.04-0.32 |

Notes S4: We examined if the effect of seed origin (local or foreign) on the number of inflorescences varied between 12 and 24 months using a Split-Plot Repeated Measures ANOVA. Origin, time and origin x time were fitted as main effects in the fixed model, while population, maternal family (nested within population), block and date were fitted in the random model. Across all population pairs there was no significant interaction between origin and time (12 and 24 months) for either of the growth or reproductive traits (*P* > 0.05, supporting material Table S4). Therefore only the growth and reproductive data for 12 months are presented.

Table S4: Repeated measures analysis to examine if the effect of plant origin (local or foreign) on progeny fitness varied between 12 and 24 months for the number of inflorescences including all population pairs. Significant model terms (*P* < 0.05) are highlighted in bold. Seed weight was included as a covariate in the analysis. A significant Origin.Time interaction term identifies variables where there was a significant difference in the effect of plant origin between 12 and 24 months.

| **Fitness component** | **df** | ***F*** | ***P* value** |
| --- | --- | --- | --- |
| **Number of inflorescences** |  |  |  |
| Seed weight | 1, 4076 | 1.33 | 0.248 |
| Origin | 1, 4076 | **4.95** | **0.026** |
| Time | 1, 4076 | **45.26** | **<0.001** |
| Origin.Time | 1, 4076 | 1.95 | 0.163 |
|  |  |  |  |
|  |  |  |  |
|  |  |  |  |
|  |  |  |  |

Table S5: STRUCTURE results to determine the most likely number of genetic clusters (*K*) following Evanno et al. (2005) for *Rutidosis leptorrhynchoides.* Δ*K* is greatest at *K* = 3, indicating that this is the most likely number of clusters.

| ***K*** | **Mean Ln Prob** | **SD** | ***K*** |
| --- | --- | --- | --- |
| 1 | -18964.96 | 0.2302 |  |
| 2 | -18639.12 | 112.8515 | 0.0992 |
| **3** | **-18302.12** | **6.3578** | **17.1914** |
| 4 | -18074.4 | 21.5918 | 0.1899 |
| 5 | -17842.6 | 17.9689 | 3.3168 |
| 6 | -17670.4 | 53.4855 | 0.1066 |
| 7 | -17503.86 | 32.1520 | 0.2581 |
| 8 | -17345.58 | 25.9876 | 0.6657 |
| 9 | -17204.65 | 26.2232 | 0.7093 |
| 10 | -17045.08 | 12.4805 | 9.7512 |
| 11 | -17007.18 | 33.2891 | 1.0093 |
| 12 | -16935.72 | 7.8719 |  |

**References**
